# Water and electricity consumption patterns with effluent quality in the textile processing industry of Bangladesh

**DOI:** 10.1039/d5ra05917h

**Published:** 2025-11-26

**Authors:** Sayam Sayam, Nayan Das, Sumiya Akter, Joya saha, Asish Sarker, Ayanshu Sen, Habibullah Habibullah, Gulger Ahmed Sajib, Infanul Haque, Md. Bayezid Mia, Md. Omar Faruk, Sanchita Fatema, Md. Kayes Munshi, Pias Chandra Paul, Md. Iftekhar Haider

**Affiliations:** a Dept. of Fabric Engineering, Barishal Textile Engineering College 8200 Barishal Bangladesh mahbub.sayamm@gmail.com; b Dept. of Wet Process Engineering, Shahid Abdur Rab Serniabat Textile Engineering College 8200 Barishal Bangladesh; c Dept. of Geography and Environmental Studies, National University of Bangladesh Gazipur Bangladesh; d Dept. of Yarn Engineering, Barishal Textile Engineering College 8200 Barishal Bangladesh; e Dept. of Yarn Engineering, Shahid Abdur Rab Serniabat Textile Engineering College 8200 Barishal Bangladesh; f Dept. of Fabric Engineering, Shahid Abdur Rab Serniabat Textile Engineering College 8200 Barishal Bangladesh; g Dept. of Apparel Engineering, Barishal Textile Engineering College 8200 Barishal Bangladesh

## Abstract

The textile industry consumes substantial amounts of water and energy during processing and is a major source of environmental pollution. To assess the current situation in the world's second-largest textile-producing country, *i.e.*, Bangladesh, this study collected water and energy consumption data as well as key water-quality parameters—pH, biological oxygen demand (BOD), chemical oxygen demand (COD), temperature, total dissolved solids (TDS), dissolved oxygen (DO), and total suspended solids (TSS)—from 14 textile factories located in Gazipur, Savar, Dhamrai (Dhaka), Narayanganj, and Chittagong. In April 2025, the recorded water consumption ranged from 200 to 68 007 m^3^ and the electricity consumption from 100 to 866 692 kWh. The solar energy usage values recorded in selected factories ranged from 5 to 11 000 kWh per month. The effluent at the outlet of textile effluent treatment plants (ETPs) showed pH = 7.1–8.1, BOD = 9–57 mg L^−1^, COD = 15–210 mg L^−1^, temperature = 21.8–34 °C, TDS = 340–1920 mg L^−1^, DO = 2.1–6.5 mg L^−1^, and TSS = 10–97 mg L^−1^, which were compared with the Bangladesh Department of Environment (DoE) standards. The study further examines the environmental, economic, and health implications of untreated or inadequately treated effluents, providing critical insights for policymakers to design effective pollution control measures and promote sustainable textile production practices.

## Abstract 2

Textile wastewater contaminants persist through treatment, accumulate in aquatic ecosystems, and transfer to organisms, posing ecological harm and potential health risks to humans exposed through water and food sources.

## Graphical abstract

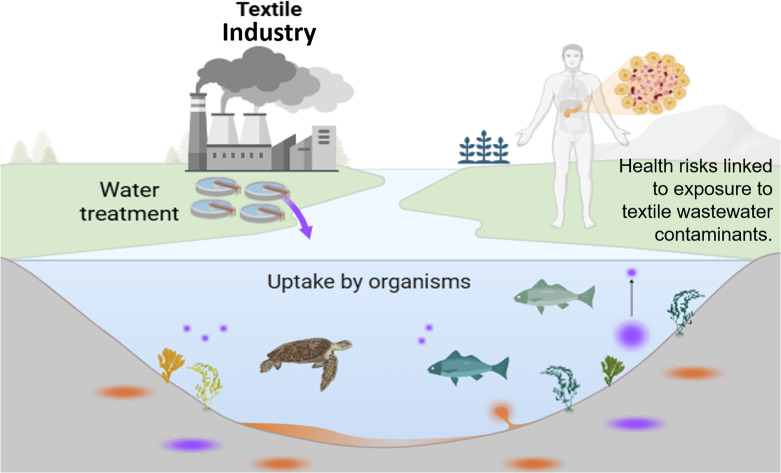

## 1. Introduction

From the beginning of the prehistoric era to today's modern world, clothing or textiles have retained an important role in human life, and it is a fundamental element of human civilization.^1^ The high rate of population growth and improved global incomes and living standards have resulted in increasing global clothing consumption, which is 400% more than the amount consumed two decades earlier.^2,3^ The textile sector is the second largest job provider across the globe and the most important sector to Bangladesh's economy, offering huge job opportunities.^4^ The global textile sector contributes approximately 6–8% of the world's GDP and represents a market valued in the trillions of dollars.^5^ In terms of manufacturing as well as exports, the textile industry of China is the largest in the world, achieving an export turnover of 288.06 billion US dollars.^6^ Moreover, India, Bangladesh, Hong Kong, Vietnam, Pakistan and Japan are notable players in textile manufacturing and export around the globe.^7^ However, the textile industry is highly water-intensive and puts a high strain on the global water resource.^8^ Several studies have revealed that the production of 1 kg of processed fabric consumes about 150 L of water, which generates a substantial amount of effluent.^9^Table 1 shows the water consumption and corresponding effluent generation amounts in various textile production processes.

**Table 1:** Water consumption and corresponding effluent generation in various textile production processes^28^

Production of the mill (m per day)	Water consumption (kL per day)	Volume of effluent (kL per day)
220 000	13 870	8000
190 000	2300	1900
80 000	3500	3400
45 000	1830	1750
35 000	1050	800

Over the past decades, groundwater decline has become a major threat to the Greater Dhaka city and its adjacent industrial zones, as the water level is decreasing at an alarming rate, potentially causing environmental degradation and drinking water scarcity.^10,11^ The textile industry is a significant contributor to this issue, as it uses large volumes of water and chemicals, making its effluents highly heterogeneous and difficult to treat.^12–14^ Untreated wastewater from textile facilities often contains varying concentrations of organic and inorganic particles depending on the operation type, and since many small-scale industries cannot afford treatment costs, they discharge directly into nearby water bodies, degrading both surface and groundwater quality.^15,16^ In terms of industrial capacity, although it is estimated that about 1700 wet processing units (including dyeing, washing, and finishing units) operate in Bangladesh, evidence suggests that the actual number is closer to 500–700, including standalone garment washing units. Moreover, only 61% of these facilities reportedly have ETPs, and among them, just 29% are compliant, while 11% to 51% are poorly designed or operated.^17^ In addition to water usage, textile production demands substantial energy; for example, producing one knitted garment from dyed-finished fabric consumes between 0.78 MJ and 1.44 MJ per unit, with additional energy required for embroidery and screen printing.^18^ Consequently, the environmental impact of the textile supply chain is enormous, contributing about 1.7 billion tons of CO_2_ annually, which accounts for nearly 10% of global greenhouse gas emissions.^5^ Growing public concern over these issues has resulted in the closure of some small-scale industries, while increased awareness worldwide has driven interest in eco-friendly techniques for wet processing.^15,19^ In Bangladesh, the Environmental Conservation Rules (1997) classify industrial units into four categories: Green, Orange A, Orange B, and Red, with fabric dyeing and chemical treatment industries falling under the Red category and requiring an Environmental Clearance Certificate before operation.^20^ Despite these concerns, most sustainability assessments of the textile industry have focused on a single parameter or individual factories. The majority of studies emphasize wastewater management, some address water consumption,^21,22^ and a few consider energy use,^23,24^ while only a few studies integrate water consumption, wastewater characteristics, and energy use in a comprehensive approach.^25–27^

With this background, the present study aims to analyze water consumption, electricity consumption, and wastewater parameters in textile industries of Bangladesh. Additionally, this paper explores environmental and economic impacts, water-saving opportunities using advanced water-analysis tools, water conservation through wastewater reuse, sustainable energy consumption strategies, and chemical innovations for eco-friendly processing.

## 2. Impact of textile effluents on human health

**Fig. 1:**
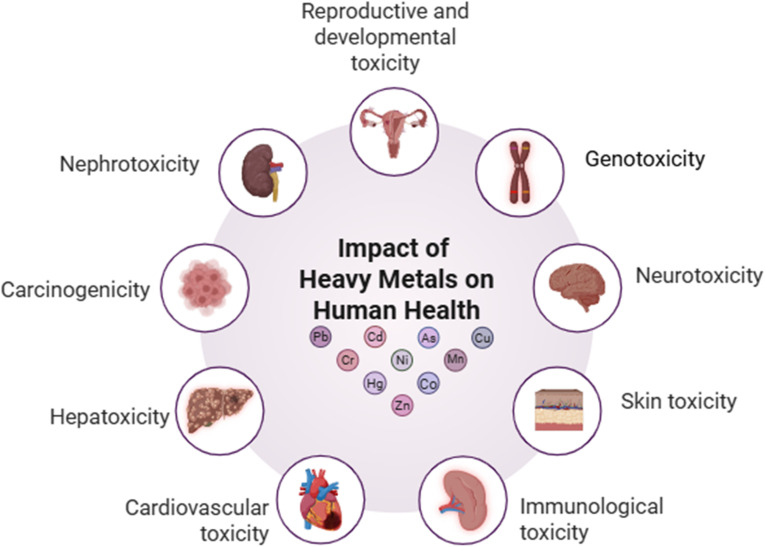
Impact of heavy metals on human health.

Each year, the textile sector releases approximately 280 000 tons of refractory textile dyes,^29^ along with more than 100 000 different dyes and 2000 types of chemicals, leading to a total textile dye output exceeding 500 000 tons.^30^ This heavy use of chemicals has serious implications for human health. The discharge of untreated or partially treated textile wastewater into surrounding environments has been linked to skin and eye irritation, corneal and conjunctival lesions, chronic dermatitis, and in some cases involving prolonged exposure, cancer.^31^ Additionally, organic pollutants, dyes, and heavy metals from textile effluents have been found to cause DNA damage and genetic mutations, as illustrated in Fig. 1.^32^ To better illustrate the connection between textile effluents and human health, Table 2 provides a detailed breakdown of specific pollutants generated during different textile wet processing steps and their associated health effects.

**Table 2:** Chemical usage and associated human health risks across different stages of textile processing^32^

Textile process	Chemical used	Pollutant nature	Health effects
Sizing	Starch, waxes, carboxymethyl cellulose (CMC), polyvinyl alcohol (PVA) and wetting agents	High in biological oxygen demand (BOD) and chemical oxygen demand (COD)	Affects the central nervous system, is carcinogenic and mutagenic
Desizing	Starch, CMC, PVA, fats, waxes and pectin	High in BOD, COD, high suspended solids (SS) and high dissolved solids (DS)	Bloating and diarrhea, irritation of the eyes and skin
Scouring	NaOH, surfactants, soaps, fats, pectin, oils, sizes and waxes	Disinfectants, insecticide residues, NaOH, detergents, oils, knitting lubricants, spin finishes and spent solvents. Non-ionic detergents may cause bloating and diarrhea, irritation to the eyes and skin	Non-ionic detergents may cause bloating and diarrhea, irritation to the eyes and skin
Bleaching	Sodium hypochlorite, H_2_O_2_, acids, surfactants, sodium silicate (NaSiO_3_), sodium phosphate, short cotton fibres, organic stabilizers and alkaline pH	High alkalinity and high suspended solids, H_2_O_2_, stabilizers and high pH	Causing severe irritation to the respiratory tract; prolonged exposure affects the liver and kidneys, leading to death
Mercerizing	Sodium hydroxide and cotton wax	High pH, low BOD and high dissolved solids	—
Dyeing	Dyestuffs, urea, reducing agents, surfactants, metals, salts, oxidizing agents, acetic acid, detergents and wetting agents	Intense color, high in BOD and dissolved solids, low in suspended solids, heavy metals, salt, surfactants, organic assistants, cationic materials, color, COD, sulphide, acidity/alkalinity and spent solvents	Eye and respiratory problems
Printing	Metals, color, formaldehyde, pastes, urea, starches, gums, oils, binders, acids, thickeners, crosslinkers, reducing agents and alkali	Strong color, high in BOD, oily appearance, suspended solids, slightly alkaline, urea, solvents, colour, metals, heat and foam	Harmful health hazards
Finishing	Softeners, solvents, resins and waxes	COD, suspended solids, toxic materials and solvents	Suppression of the hematological system

In a broader context, textile-related pollution exacerbates water security issues in developing countries. For instance, according to the World Health Organization (WHO), 2 billion people worldwide still lack access to safe drinking water.^33^ In polluted environments, humans are exposed to chemical contaminants not only through ingestion but also *via* inhalation and skin contact. Chemicals present in ambient air around factories can be inhaled, while the skin directly absorbs pollutants during routine exposure.^34^ Furthermore, heavy metals, such as lead and cadmium, used in textile manufacturing are non-biodegradable and tend to bioaccumulate in the human body over time, increasing the risk of chronic health conditions.^35^ Exposure also occurs through indirect pathways, such as the food chain. Contaminated irrigation water leads to chemical uptake by crops, eventually entering the human diet.^36^

Empirical findings support these risks. A 2023 questionnaire survey involving 200 residents living near 860 small dyeing units found that 84% of respondents believed their family's health had declined since the factories began operation, reporting skin rashes, asthma, and typhoid as the most common conditions.^37^ Similarly, Li *et al.*^38^ reported a high median concentration of phthalate esters (PAEs) at 4.15 mg g^−1^ in infant cotton clothing, identifying clothing as a significant source of dermal exposure, greater than air or dust. In contrast, Zhang *et al.*^39^ observed lower PAE levels in new face towels in China, with a median of 0.000173 mg g^−1^, though higher concentrations were found in used towels and coral velvet fabrics than in cotton.

## 3. Environmental impacts of textile processing

The textile industry's substantial energy consumption has significant environmental consequences.^1^ Textiles contribute 5–10% of global greenhouse gas (GHG) emissions.^40^ With the current consumer demand trends, the global consumption of textiles and apparel is expected to triple by 2050.^41,42^ This increase could represent nearly 26% of the total carbon budget required to stay within the 2 °C global warming limit.^43^ The World Resources Institute estimates that 1.025 gigatonnes (Gt) of carbon dioxide equivalent (CO_2_), or roughly 2% of annual global GHG emissions, was emitted in 2019.^44^ In 2015, the CO_2_ and ammonia nitrogen emissions from Chinese textile enterprises were 94.71 billion tons and 14 823 tons, which accounted for 6.02% and 7.55% of the total emissions from all industrial enterprises, respectively.^45^ The Vietnam textile industry emits approximately 5 million tons of CO_2_ per year.^46^ In a study of the Pakistani textile industry, the carbon footprint in 2010 was 20 436.615167 tCO_2_, while in 2014, it was nearly 42 867.72 tCO_2_, indicating that emissions almost doubled over the five years.^47^ The carbon emissions of the Bangladesh textile industry increased more than 10 times between 1983 and 2019. While the carbon emission was 8236 metric tons in 1983, it increased to 93 761 metric tons in 2019.^48^Table 3 shows the energy-related carbon dioxide emissions (estimates) of major textile and garments exporting countries.

**Table 3:** CO_2_ emissions from textile industries by country and year

Study year	Country/organization	Total CO_2_ emissions from textile industries	Ref.
2015	China	94.71 billion metric tons of CO_2_	45
2019	Bangladesh	93 761 metric tons of CO_2_ equivalent per year	47
2021	Germany	38 million metric tons of CO_2_ equivalent per year	49
2022	European Union	121 million metric tons of CO_2_ equivalent per year	48
2023	Pakistan	8.1 million metric tons of CO_2_ equivalent per year	50
2024	India	65 million metric tons of CO_2_ equivalent per year	51
2024	Vietnam	5 million metric tons of CO_2_ equivalent per year	46

At the same time, water resource pollution has emerged as a critical issue due to the textile industry's large water consumption.^45^ Consequently, water scarcity stands out as one of the most significant sustainability challenges confronting the textile industry today.^52^ For instance, Dhaka City has experienced a dramatic decline in groundwater levels, with over 200 feet of depletion recorded in the past 50 years, and this downward trend is continuing.^53^ In addition to the sheer volume of water consumption, the resulting generation of wastewater poses a significant ecological threat, primarily due to the presence of various harmful chemicals, including dyes, heavy metals, and surfactants.^54^ Furthermore, Table 4 summarizes the ecological consequences associated with wastewater generated by the textile industry.^55^ Notably, textile production is estimated to contribute approximately 20% of global clean water pollution, primarily through dyeing and finishing processes.^56^ One primary reason for this is the inefficiency of dyeing operations, which results in a substantial loss of dyestuffs that enter the effluent and eventually discharge into natural water systems, gradually degrading both surface and groundwater quality.^57^ Additionally, the discharge of effluents at high temperatures exacerbates the problem by reducing dissolved oxygen concentrations in water bodies, severely impacting aquatic ecosystems.^58^ To illustrate the severity of the issue, China serves as a striking example, where nearly 70% of rivers, lakes, and reservoirs are affected by pollution, with its textile industry comprising over 50 000 mills being a major contributor.^59^

**Table 4:** Environmental impacts and associated pollutants with load factors per kg of textile material^55^

Environmental impact	Pollutant type	Characterization factor
Eutrophication	COD	0.022 kg of pollutant per kg of material
	BOD	0.11 kg of pollutant per kg of material
	NH_3_–N	3.64 kg of pollutant per kg of material
Acidification	H_2_SO_4_	0.65 kg of pollutant per kg of material
	Chloride	0.88 kg of pollutant per kg of material
Alkalization	NaOH	0.425 kg of pollutant per kg of material
	Na_2_CO_3_	0.321 kg of pollutant per kg of material
Ecotoxicity	Zn^2+^	0.38 m^3^ of affected water per kg of pollutant
	CS_2_	0.18 m^3^ of affected water per kg of pollutant

## 4. Methodology

### 4.1 Study area

This study was conducted within Bangladesh's major textile manufacturing zones, specifically Gazipur, Savar, Dhaka, Narayanganj, and Chittagong, which are recognized as the country's largest hubs for apparel and textile processing. A total of 14 textile factories were selected for comprehensive data collection. Six factories were chosen from Gazipur (labeled B, E, F, H, K, and L), five from Savar (labeled A, C, D, G, and N), and one each from Dhamrai (Dhaka), Narayanganj, and Chittagong (labeled I, J, and M, respectively). The sample included a mix of small-, medium-, and large-scale factories to capture variations in water consumption, energy usage, and wastewater quality parameters across different production capacities. Among the selected facilities, some were certified green factories, enabling a comparative analysis between sustainable and conventional manufacturing practices. These industrial clusters are located near major river systems that are critically impacted by industrial discharges. Previous studies have reported severe contamination of these rivers with heavy metals, dyes, and other organic pollutants, posing significant risks to aquatic ecosystems and public health.^60^

### 4.2 Factory selection criteria

The research team consisted of members who were completing their internships in various textile industries during the data collection period, which provided direct access to operational areas and enhanced data accuracy. As part of their internship requirements, each member spent approximately 15 days working at effluent treatment plants (ETPs) and monitoring water and electricity consumption at designated stations, thereby gaining practical experience relevant to this study. A purposive sampling method was employed to ensure diversity in the sample, selecting factories of different scales, including small, medium, and large, to capture variations in water and energy use, as well as wastewater characteristics across different operational capacities. Initially, 20 factories were approached through formal communication facilitated by the team members stationed at those facilities; however, 6 factories declined participation due to internal regulations and confidentiality concerns. Ultimately, data were successfully collected from 14 factories that agreed to provide information and participate in interviews.

The selected factories, summarized in Table 5, represent a diverse range of product types and operational capacities within Bangladesh's textile sector. Most factories operate bio-chemical ETPs, while a few have incorporated membrane bioreactor (MBR) systems or achieved Green Factory certification. This variation provides a balanced representation of both conventional and environmentally advanced facilities, enabling comparative analysis of water and energy consumption, effluent treatment efficiency, and sustainability performance across different factory types.

**Table 5:** Characteristics of the selected textile factories, including product pypes, effluent treatment technologies, and Green Factory classification

Factory	Main product types	Primary ETP technology	Green Factory status
A	Polo shirts, T-shirts, tank tops, trousers, hooded jackets, cardigans, sportswear, undergarments	Bio-chemical	Not Green Factory
B	Denim, non-denim, jackets, dresses	Bio-chemical	Green Factory
C	Polo shirts, T-shirts, tank tops, trousers, hooded jackets, cardigans, sportswear, undergarments, lightly woven	Bio-chemical	Green Factory
D	Woven, denim, non-denim, jackets, dresses, polo shirts, T-shirts, tank tops, trousers, hooded jackets, cardigans, sportswear, undergarments, lightly woven	Bio-chemical	Green Factory
E	Polo shirts, T-shirts, tank tops, trousers, hooded jackets, cardigans, sportswear, undergarments	Bio-chemical	Not Green Factory
F	Polo shirts, T-shirts, tank tops, trousers, hooded jackets, undergarments	Bio-chemical	Not Green Factory
G	Woven and nonwoven garments	Bio-chemical	Green Factory
H	Polo shirts, T-shirts, tank tops, trousers, hooded jackets	Bio-chemical + membrane bioreactor (MBR)	Not Green Factory
I	Polo shirts, T-shirts, tank tops, trousers, hooded jackets	Bio-chemical	Not Green Factory
J	Polo shirts, T-shirts, tank tops, trousers, hooded jackets, cardigans, sportswear, undergarments	Bio-chemical	Green Factory
K	Polo shirts, T-shirts, tank tops, trousers, hooded jackets, sportswear	Bio-chemical + membrane bioreactor (MBR)	Green Factory
L	Polo shirts, T-shirts, tank tops, trousers, hooded jackets, sportswear	Bio-chemical	Not Green Factory
M	Polo shirts, T-shirts, tank tops, trousers, hooded jackets, cardigans, sportswear	Bio-chemical	Not Green Factory
N	Polo shirts, T-shirts, tank tops, trousers	Bio-chemical	Green Factory

### 4.3 Data collection and analysis

Groundwater consumption data were collected from the Head or representative of the Utility Department, electricity consumption data from the Head of Production, and wastewater-related data from the Head of the ETP. While some factories provided the complete dataset as requested, others shared only partial information, possibly due to unavailability or confidentiality concerns. All of the data were collected on April 15, 2025. All collected data were organized and stored using Google Docs for efficient management and collaborative verification. Descriptive statistical techniques were applied for analysis, where categorical variables were presented as percentages, and continuous variables were summarized using means and standard deviations to provide a comprehensive understanding of the water and energy consumption patterns and wastewater quality characteristics. Principal Component Analysis (PCA) was also applied to group parameters with comparable characteristics. This technique converts the original variables into principal components (PCs), which are linear combinations that represent the variance in the dataset.^61^ Loadings close to ±1 signify a strong influence on the variables, whereas those near zero indicate a minimal effect.^62^

## 5. Results and discussion

### 5.1 Water and energy consumption patterns

Table 6 provides the water and energy consumption in each factory in a day. This research found that the energy consumption varied between 100 to 866 692 kWh. Similarly, the water consumption ranged from 200 and 68 007 m^3^. To enable a meaningful comparison among factories of different production capacities, daily production data (in kg of fabric) were collected and used to calculate water consumption (WC, m^3^ kg^−1^) and energy consumption (EC, kWh kg^−1^). The results show that the WC values ranged from 0.09 to 4.92 m^3^ kg^−1^, while EC ranged from 0.39 to 2.18 kWh kg^−1^. Large-scale factories, such as M (Chittagong) and L (Gazipur), exhibited lower specific consumption, indicating higher operational efficiency, whereas smaller factories, such as A and N, showed higher values, reflecting limited recycling and older technology.

**Table 6:** Water and energy consumption per kg of textile production in factories

Factory	Production (kg per day)	Water (m^3^ per day)	Electricity (kWh per day)	Water (m^3^ per kg)	Energy (kWh per kg)
A	253.7	200	100	0.79	0.39
B	45 120	9000	45 100	0.20	1.00
C	4486	5000	8900	1.12	1.98
D	5039	3200	6000	0.64	1.19
E	49 236	10 270	40 500	0.21	0.82
F	34 268	7300	48 200	0.21	1.41
G	3574	2450	5600	0.69	1.57
H	27 300	6500	48 900	0.24	1.79
I	30 482	5200	50 515	0.17	1.66
J	26 850	2377	58 526	0.09	2.18
K	40 190	8500	55 900	0.21	1.39
L	94 870	9800	185 000	0.10	1.95
M	410 586	68 007	866 692	0.17	2.11
N	6138	30 193	7470	4.92	1.22
Average ± SD	—	—	—	0.62 ± 1.27	1.49 ± 0.55

In order to contextualize the regional performance, a 2019 study conducted in Fakir Fashion Ltd, Narayangonj revealed that the industry was highly water-intensive, with monthly groundwater extraction volumes ranging from 128 865 to 193 003 m^3^.^27^ In the same area, this study revealed that factory J consumed 2377 m^3^ of water per day, which is significantly lower than the earlier finding. This suggests that factory J may be implementing stricter water control measures in line with updated regulatory policies. In addition, this industry is actively engaged in several sustainability initiatives, including participation in ‘Switch to Circular Economy’ and other water-efficiency programs. These projects have contributed to improved process optimization, wastewater reuse, and responsible resource management, which collectively explain the substantial reduction in groundwater extraction without compromising production capacity.

Building on this, a region-wise comparison reveals that factories in Gazipur demonstrated the highest average electricity consumption, with six factories (B, E, F, H, K, L) using an average of approximately 49 562 kWh per day. Within this group, factory L stood out with a daily usage of 185 000 kWh, which alone is nearly 3.7 times higher than the combined average of the other five Gazipur factories. In contrast, Savar-based factories (A, C, D, G, N) reported a much lower average electricity consumption of approximately 6332 kWh per day. Thus, Gazipur's energy usage is nearly 8 times greater than Savar's, indicating either higher production volumes, more machinery, or lower energy efficiency. The factory in Dhamrai (factory I) consumed 50 515 kWh, which is comparable to the Gazipur average and significantly higher than Savar's. Similarly, Narayangonj's factory (J) consumed 58 526 kWh, around 17% higher than Dhamrai, while Chittagong's factory (M) consumed 866 692 kWh, a value over 17.5 times greater than the entire Gazipur average and more than 14 times higher than the combined electricity consumption of all factories except M.

In terms of water consumption, Chittagong again ranked highest with 68 007 m^3^ per day, which represents 41.8% of the total daily water consumption recorded across all factories. Gazipur-based factories reported an average daily water usage of 6964 m^3^, with factory E consuming the most in the region at 10 270 m^3^ per day, followed by factory B at 9000 m^3^. Savar, in contrast, had an average water consumption of 7308 m^3^ per day among five factories, but this number is heavily influenced by factory N, which alone consumed 30 193 m^3^. Excluding this outlier, the average water use in Savar drops to 2337 m^3^ per day, making it about 66% lower than the Gazipur average. Factory I in Dhamrai consumed 5200 m^3^ daily, while Narayangonj's factory (J) used 2377 m^3^, placing both in a moderate range. These findings indicate that Chittagong and Gazipur are the most resource-intensive regions in terms of both energy and water. Savar displays the lowest average consumption overall, suggesting either smaller-scale operations or relatively more efficient resource management, particularly when factory N is excluded from the average. Additionally, Dhamrai and Narayangonj represent moderate consumption profiles, though their electricity usage aligns more closely with the higher-consuming Gazipur region, while their water usage remains modest.

When the data were normalized by the production volume, however, a clearer efficiency pattern emerged. Large-scale factories, such as M (Chittagong) and L (Gazipur), exhibited the lowest specific water and energy consumptions, 0.10–0.17 m^3^ kg^−1^ and 1.95–2.11 kWh kg^−1^, respectively, demonstrating higher operational efficiency. In contrast, smaller units, such as A and N, recorded much higher unit consumptions, up to 4.92 m^3^ kg^−1^ and 1.22 kWh kg^−1^, reflecting the influence of older machinery, batch-wise processes, and limited recycling. Mid-scale factories (B–K) fell between these extremes, indicating that economies of scale, technological modernization, and improved process integration substantially contribute to lower specific resource use. Further analysis was conducted to examine whether “Green Factories” demonstrate superior resource efficiency compared to conventional ones. Based on the normalized data (Table 5), certified green factories (B, C, D, G, J, K, N) showed an average water consumption of 0.64 ± 0.27 m^3^ kg^−1^ and energy consumption of 1.38 ± 0.40 kWh kg^−1^, while non-green factories averaged 0.60 ± 1.12 m^3^ kg^−1^ and 1.57 ± 0.63 kWh kg^−1^, respectively. Although the differences are moderate, green factories generally maintain tighter control over water and energy use due to improved process monitoring and wastewater recycling facilities. However, the overlap in consumption ranges indicates that certification alone does not automatically ensure higher efficiency; technological modernization and consistent operational practices remain essential to achieving sustained reductions.

### 5.2 Wastewater quality analysis

#### 5.2.1 pH

**Fig. 2:**
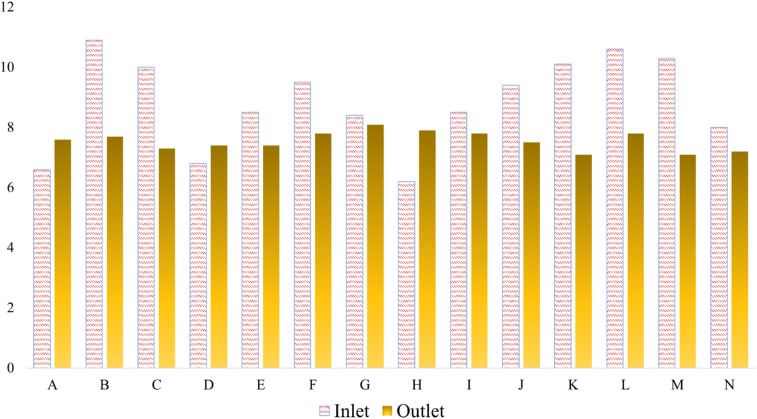
pH values at the inlet and outlet of the textile ETP.

Maintaining proper pH levels in textile effluents is critical for environmental protection and regulatory compliance. Fig. 2 shows the inlet and outlet pH levels across the textile factories included in this research. According to the Department of Environment (DoE), Bangladesh, the discharge pH of treated wastewater should be between 6 and 9. In the Gazipur region, this study found that influent pH values ranged from 6.2 to 10.9 and outlet values from 7.1 to 7.9. This variation indicates differences in the efficiency of neutralization and aeration processes across factories. For instance, factory B had the highest inlet pH of 10.9 and discharge pH of 7.7, while factory H had the lowest inlet value of 6.2 and discharge at 7.9. Factory H uses a hybrid biological-chemical ETP system, which helps stabilize pH through biological oxidation and balanced chemical dosing. These findings correspond to those of Ahmad *et al.*,^63^ who reported outlet pH values ranging from 7.4 to 8.4 in the same area. Similarly, Rafiq *et al.*^64^ found outlet pH values of 7.2 and 7.6 in Gazipur, aligning well with values observed in factories F (7.8), K (7.1), and L (7.8). Factories F and K operate membrane bioreactor and biological treatment units that maintain near-neutral discharge pH values by improving buffer capacity and reducing chemical dependency. Conversely, another study in Gazipura, Gazipur reported a discharge value of 6.4,^65^ which is lower than all outlet values found in this study, indicating possible variations in treatment performance across sub-regions. Such deviations are often linked to conventional treatment setups with less precise chemical dosing and minimal automation, resulting in pH fluctuations.

In the Savar region, this research observed inlet pH values ranging from 6.6 to 10.0 and outlet values from 7.2 to 8.1. Here as well, variation reflects differences in operational control, where factories with automated dosing and continuous aeration achieved more consistent results. Notably, factory C had the highest inlet pH at 10.0 and reduced it to 7.3, whereas factory A had the lowest inlet value at 6.6, resulting in an outlet of 7.6. This performance improvement is likely due to proper monitoring and balanced reagent application within the ETP units. These results are slightly higher than the findings of a study by ref. 66, which recorded discharge values between 6.4 and 7.0 in Savar, where the Fenton oxidation process was used. In Dhamrai, factory I reduced pH from 8.5 to 7.8, aligning closely with outlet values in Gazipur. Factory I uses a conventional physical–chemical process, explaining its moderate reduction efficiency compared with those of hybrid systems. In Narayangonj, factory J showed a reduction from 9.4 to 7.5, which corresponds with the reports of Uddin *et al.*,^67^ who also reported a discharge value of 7.5 in that area. Finally, in Chittagong, factory M recorded an inlet pH of 10.3 and an outlet of 7.1. This factory employs a chemical neutralization unit that effectively adjusts pH but may result in slightly higher residual variability. Piyash *et al.*^68^ found a discharge pH of 6.5 in Chittagong, which is 0.6 units lower than the present study's result. In contrast, another study reported values between 8.0 and 8.5,^69^ indicating that this study's result falls just below that earlier range.

#### 5.2.2 BOD

According to the DoE, the allowable Biological Oxygen Demand (BOD) for textile effluent discharge is 30 mg L^−1^. In this study, most textile factories maintained this standard. However, factory K in Gazipur exhibited a BOD discharge of 59.5 mg L^−1^, nearly twice the DoE limit. While this value is high, it remains lower than those of three other Gazipur-based factories reported in a previous study, which recorded outlet BOD values of 120, 153, and 259 mg L^−1^ across five surveyed industries.^63^ The remaining two industries in that study were within the permissible range. Additionally, factory K showed the highest influent BOD of 488 mg L^−1^ among all 14 factories in this research, likely due to heavy chemical usage in wet processing and finishing. In contrast, factory L from the same region maintained the lowest outlet BOD at 14 mg L^−1^, demonstrating substantial compliance with the discharge standards. Furthermore, another study in two Gazipur factories reported BOD discharge levels of 42.2 and 58 mg L^−1^, respectively,^63^ both of which are slightly higher than most values observed in this study.

**Fig. 3:**
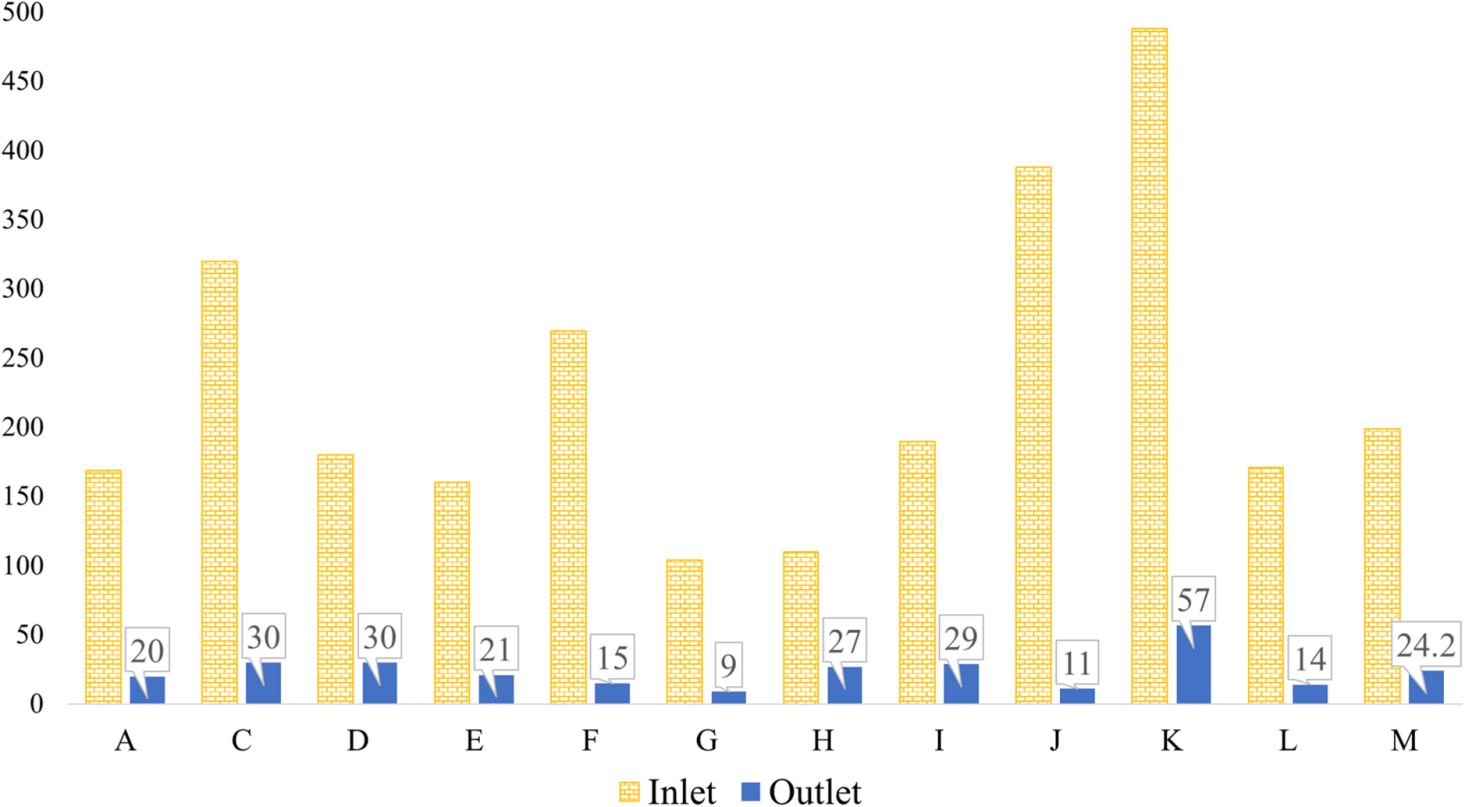
BOD levels at the inlet and outlet of the textile ETP.

In Savar, which lies adjacent to Gazipur within the Dhaka region, the highest influent BOD was recorded at 320 mg L^−1^ in factory C, as shown in Fig. 3. This factory effectively reduced its BOD to the exact DoE limit of 30 mg L^−1^. Remarkably, factory G—focused primarily on washing and dyeing—achieved the lowest outlet BOD in Savar. Their investment in process efficiency and water reduction technologies appears to be reflected in their effective ETP performance. A 2022 study covering six factories in the same area found that all discharge values exceeded the levels found in this current research.^66^ In Dhamrai, factory I recorded an influent BOD of 190 mg L^−1^ and an outlet of 29 mg L^−1^, which not only meet the national standards but are also significantly lower than the outlet values reported in recent research by Rafiq *et al.*^64^ In Narayangonj, although the influent BOD of factory J was comparatively high, suggesting intensive chemical use, its outlet value remained well below 30 mg L^−1^, indicating a highly efficient treatment process. In Chittagong, factory M discharged BOD at 24.2 mg L^−1^, remaining within the acceptable limit. This aligns closely with findings by Dipu and Piyash (2024), who reported a notably low BOD of 9 mg L^−1^ in that region.^70^ On the other hand, a 2022 study showed that three factories in Chittagong had elevated BOD values, though one factory reported a favorable discharge of 29 mg L^−1^,^69^ slightly above that of factory M in this study. Altogether, these comparisons underscore that while variations exist, the majority of factories in this study demonstrated better or comparable performance to earlier reports.

#### 5.2.3 COD

**Fig. 4:**
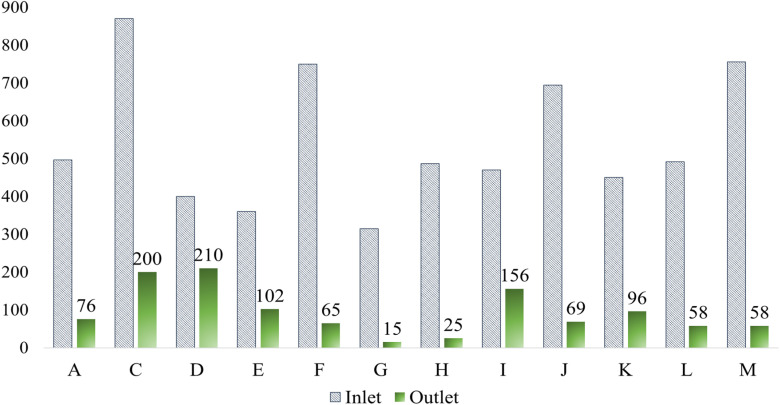
COD levels at the inlet and outlet of the textile ETP.

This study revealed that the maximum inlet and outlet COD levels were 870 mg L^−1^ and 200 mg L^−1^, respectively, observed in factory C from Savar. Interestingly, the lowest outlet COD was also found in Savar (15 mg L^−1^ in factory G), as depicted in Fig. 4. The average outlet COD level across the Savar factories in this study was 125.25 mg L^−1^. In comparison, previous studies reported significantly higher values in the same region, with an average outlet COD of 298.62 mg L^−1^, encompassing individual values, such as 570, 280, 170, and 160 mg L^−1^.^66^ This indicates a notable 58.06% reduction in COD levels, suggesting improved effluent treatment performance through better chemical usage and operational efficiency. In Gazipur, five factories recorded outlet COD levels ranging between 25 and 102 mg L^−1^, with an average of 69.2 mg L^−1^, all well within the DoE limit of 200 mg L^−1^. In contrast, a recent study in Gazipur showed considerably higher COD values—465, 305, 357, 91, 85, and 80 mg L^−1^—averaging 230.5 mg L^−1^.^63^ This reflects a 69.97% decrease in average COD levels compared to the past, indicating significant advancements in treatment practices and regulatory compliance in the region. In the case of Narayangonj, factory J exhibited an outlet COD value of 69 mg L^−1^, while earlier research from 2024 reported outlet values of 519, 511, 490, 277, and 133 mg L^−1^, averaging 386 mg L^−1^.^64,71^ Moreover, in Dhamrai, Dhaka, the outlet COD in factory I was found to be 156 mg L^−1^, still under the regulatory limit but higher than the Gazipur and Narayangonj values, indicating potential scope for optimization. Factory M in Chittagong showed strong performance with an outlet COD of 58 mg L^−1^, aligning well with regulatory standards and recent literature, which reported outlet COD values as low as 36 mg L^−1^.^70^

#### 5.2.4 Temperature

**Fig. 5:**
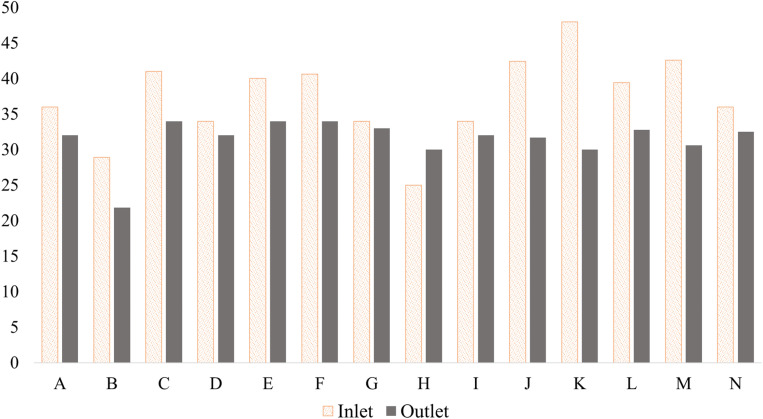
Inlet and outlet temperature of the textile ETP.

According to the DoE, the discharged water must not exceed more than 5 °C above the temperature of the receiving water body. From Fig. 5, it is evident that the outlet wastewater temperature of all industries decreased compared to the inlet temperature, with all values remaining within DoE policy limits, thereby ensuring regulatory compliance. A review study on textile wastewater in Bangladesh reported temperatures ranging from 25 °C to 45 °C in the Narayangonj area.^28^ The present findings for Narayangonj (factory J) also fall within this range. The lowest outlet temperature was observed in factory B (Gazipur), showing a reduction of 7.1 °C from inlet to outlet, mainly due to its already low inlet value, suggesting less heat load from production. In Savar, the highest outlet temperature was 34 °C, recorded in factories C and E, reduced from inlet temperatures of 41 °C and 40 °C, respectively. For Savar, the outlet temperature averaged 32.3 °C with a standard deviation of 1.5 °C, indicating consistent thermal control, likely supported by effective cooling systems and increased wastewater retention time. A study in Chittagong involving six textile factories found maximum and minimum temperatures of 38 °C and 22 °C, respectively, with a standard deviation of 4.41 °C.^72^ In the present study, Chittagong's outlet temperature was 30.6 °C, much lower than the reported maximum, which may be due to more efficient heat exchange processes in ETP.

#### 5.2.5 TDS

**Fig. 6:**
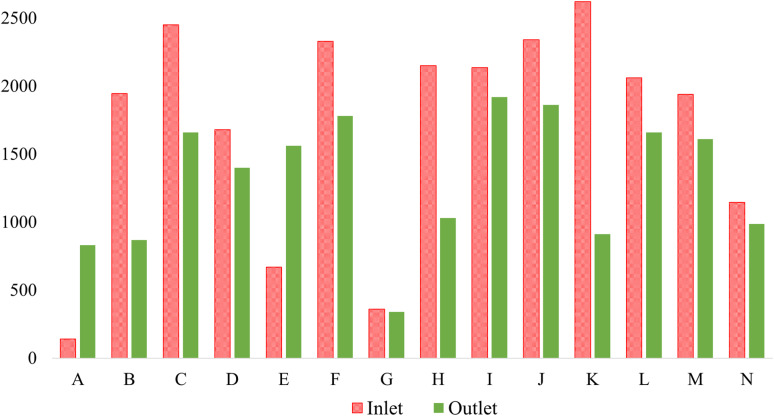
TDS levels at the inlet and outlet of the textile ETP.

The inlet water samples represent wastewater generated mainly from dyeing, washing, bleaching, and finishing units of the studied factories, as these stages collectively contribute the highest dissolved solids due to the use of salts, alkalis, and surfactants. Total Dissolved Solids (TDS) in textile effluent is a key indicator of dissolved inorganic and organic matter, directly linked to salinity and conductivity levels. High TDS concentrations can adversely affect aquatic life, alter soil permeability, and reduce the suitability of water for reuse. The DoE in Bangladesh recommends a maximum discharge limit of 2100 mg L^−1^ for TDS in textile effluent. In this study, inlet TDS concentrations ranged from 140 mg L^−1^ to 2620 mg L^−1^, while outlet concentrations varied between 340 mg L^−1^ and 1920 mg L^−1^, as illustrated in Fig. 6. Location-wise analysis showed that the average inlet TDS concentration in Savar, where five factories were studied, was approximately 1215 mg L^−1^, with a standard deviation of 862 mg L^−1^, and the average outlet concentration was about 1043 mg L^−1^, with a standard deviation of 484 mg L^−1^. In Gazipur, with six factories included, the average inlet TDS was 1964 mg L^−1^, with a standard deviation of 632 mg L^−1^, and the outlet average was 1284 mg L^−1^, with a standard deviation of 379 mg L^−1^. Dhaka's Dhamrai area had a single facility with an inlet value of 2135 mg L^−1^ and an outlet of 1920 mg L^−1^. Narayanganj's factory showed an inlet TDS of 2340 mg L^−1^ and an outlet of 1860 mg L^−1^, while Chittagong reported 1940 mg L^−1^ for the inlet and 1611 mg L^−1^ for the outlet. The lowest inlet concentration was recorded at a small-scale factory in Savar, while the highest inlet value was found in Gazipur. Despite these variations, the highest outlet concentration, recorded in Dhamrai, remained below the regulatory limit and was much lower than those reported in previous studies. For comparison, earlier TDS ranges of 1637 to 6170 mg L^−1^ by Kamal *et al.*,^73^ 1056 to 7130 mg L^−1^ by Nergis *et al.*,^74^ 518 to 2240 mg L^−1^ by Ahmad *et al.*,^63^ and 1556 to 1996 mg L^−1^ by Rafiq *et al.*^64^ were documented, demonstrating that the current TDS levels in this study are generally lower than previous values. The variation in the outlet TDS among factories is largely associated with differences in their treatment technologies and operational efficiency. Factories using reverse-osmosis or advanced hybrid ETPs showed significant TDS reduction through ion removal and water-reuse systems, while conventional physical-chemical units sometimes exhibited higher outlet TDS due to chemical dosing and sludge recirculation.

#### 5.2.6 DO

**Fig. 7:**
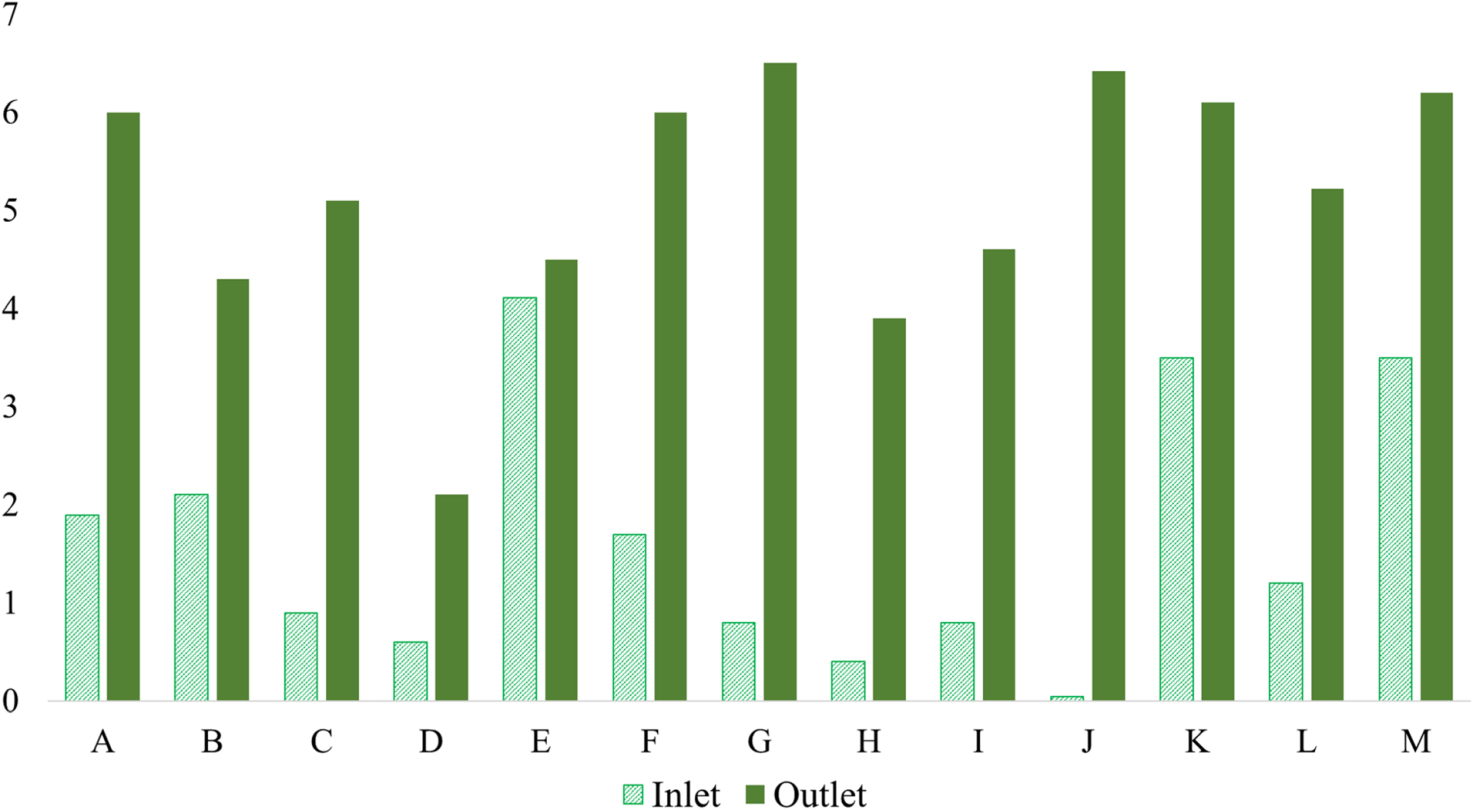
DO levels at the inlet and outlet of the textile ETP.

Fig. 7 presents the DO levels observed in this study, illustrating both inlet and outlet concentrations across various textile factories. The inlet DO values ranged widely from as low as 0.04 mg L^−1^ in Narayanganj (factory J) to 4.11 mg L^−1^ in Gazipur (factory E), while the outlet values consistently showed improvement, ranging between 2.1 mg L^−1^ and 6.5 mg L^−1^. These findings align well with previous studies, such as Roy *et al.*,^75^ who reported inlet DO values between 0.11 and 0.5 mg L^−1^; Masum *et al.*,^72^ with a broader range of 0.14 to 6.22 mg L^−1^; and Hossain *et al.*,^66^ who observed DO levels from 5.8 to 6.8 mg L^−1^ after treatment. The low DO levels observed in the inlet are primarily due to the combined wastewater entering the ETP from various production sections, including dyeing, washing, bleaching, and finishing. These process streams are rich in organic matter, dyes, surfactants, and reducing agents, such as sodium hydrosulfite and sulfites, which rapidly consume available oxygen. Furthermore, wastewater from dyeing and bleaching operations often reaches the ETP at elevated temperatures and without aeration, intensifying oxygen depletion during conveyance and mixing. Consequently, by the time the combined wastewater enters the ETP inlet, the dissolved oxygen concentrations are already significantly reduced.

Regionally, Savar exhibited inlet DO values averaging approximately 1.0 mg L^−1^, with outlet values increasing to about 4.9 mg L^−1^, demonstrating notable enhancement through effluent treatment. Gazipur factories showed a slightly higher average inlet DO of around 2.1 mg L^−1^, with outlet values averaging 4.8 mg L^−1^, reflecting adequate oxygenation and biological treatment. In contrast, Narayanganj's single observed factory had the lowest inlet DO but achieved one of the highest outlet values at 6.42 mg L^−1^, indicating a well-managed wastewater treatment system. Similarly, Chittagong's factory showed an inlet DO of 3.5 mg L^−1^ and an outlet of 6.2 mg L^−1^, consistent with effective treatment and aeration practices.

#### 5.2.7 TSS

**Fig. 8:**
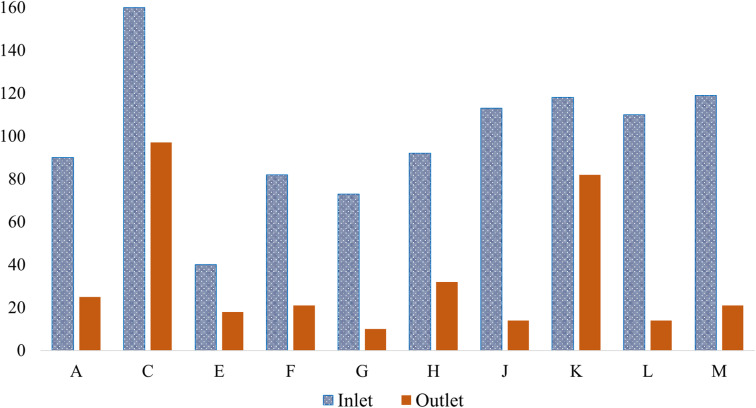
TSS levels at the inlet and outlet of the textile ETP.

TSS is a critical parameter in assessing the quality of textile wastewater, as high TSS levels can reduce light penetration in receiving waters and negatively affect aquatic ecosystems. Previous studies have reported a wide range of TSS concentrations in textile effluents, including 49 to 462 mg L^−1^ in Pakistan,^74^ 55.3 to 254.7 mg L^−1^ in Egypt,^73^ 2.69 to 0.05 mg L^−1^ (ref. 72) and 100 to 336 mg L^−1^ (ref. 75) in Bangladesh, and 35 to 1200 mg L^−1^ in Nigeria.^76^ In this study, inlet TSS values varied from 40 mg L^−1^ to 160 mg L^−1^, with the lowest inlet recorded in Gazipur (factory E) and the highest in Savar (factory C). Correspondingly, outlet TSS values ranged between 10 mg L^−1^ and 97 mg L^−1^, showing a significant reduction after treatment. Regionally, Savar factories averaged an inlet TSS of approximately 107 mg L^−1^ and outlet values around 44 mg L^−1^, indicating effective solids removal, while Gazipur factories exhibited a slightly lower average inlet TSS of about 88 mg L^−1^ and outlet values averaging 33 mg L^−1^. The Narayanganj factory reported an inlet of 113 mg L^−1^ and a remarkably low outlet value of 14 mg L^−1^, reflecting efficient wastewater treatment practices. The factory in Chittagong recorded an inlet TSS of 119 mg L^−1^ and an outlet of 21 mg L^−1^, consistent with the trend of substantial reduction in suspended solids. Compared with previous research, the TSS values observed in this study generally fall within or below the reported ranges, demonstrating adherence to treatment standards and improvements in effluent quality. Fig. 8 demonstrates the TSS levels of textile ETP.

Beyond the measured parameters, several studies have reported that textile effluents in Bangladesh and other developing countries frequently contain elevated concentrations of heavy metals, such as Cr, Pb, Cu, and Zn, as well as sulfide, phenolic compounds, and ammoniacal nitrogen.^77^ These substances originate mainly from dyeing auxiliaries, mordants, and finishing chemicals. For instance, Cr and Pb are often associated with metal-complex dyes and pigments, while phenolic compounds and sulfide arise from reduction and bleaching operations. Ammoniacal nitrogen is typically produced from the degradation of nitrogenous compounds and urea-based dyes. Elevated levels of these contaminants have been linked to aquatic toxicity, eutrophication, and bioaccumulation risks, which can ultimately impact human health through the food chain. Hence, even though these parameters were not included in the present dataset, their consideration is essential for a holistic evaluation of textile effluent impacts on water quality.

## 6. Descriptive statistics and multivariate analysis

Table 7 provides descriptive statistics for the wastewater quality parameters (pH, BOD, COD, Temp., TDS, DO, TSS) from the outlet of 14 textile factories. The mean ± SD values were 7.55 ± 0.31 for pH, 23.93 ± 12.77 mg L^−1^ for BOD, 94.17 ± 63.24 mg L^−1^ for COD, 31.46 ± 3.09 °C for temperature, 1315.14 ± 481.26 for TDS, 5.15 ± 1.27 for DO, and 33.40 ± 30.41 for TSS. The maximum and minimum values ranged as follows: pH (8.10–7.10), BOD (57–9) mg L^−1^, COD (210–15) mg L^−1^, temperature (34–21.8)°C, TDS (1920–340) mg L^−1^, DO (6.5–2.1) mg L^−1^, and TSS (97–10) mg L^−1^.

**Table 7:** Descriptive statistics of the textile effluent parameters

Parameters	pH	BOD	COD	Temp.	TDS	DO	TSS
Max	8.10	57.00	210.00	34.00	1920.00	6.50	97.00
Min	7.10	9.00	15.00	21.80	340.00	2.10	10.00
Mean	7.55	23.93	94.17	31.46	1315.14	5.15	33.40
Median	7.55	22.60	72.50	32.00	1480.00	5.22	21.00
Mode	7.80	30.00	58.00	32.00	0.00	6.00	21.00
Standard deviation	0.31	12.77	63.24	3.09	481.26	1.27	30.41
Coefficient of variation	4.15	53.37	67.15	9.81	36.59	24.57	91.06
Skewness	0.03	1.34	0.75	−2.31	−0.47	−1.01	1.40
Kurtosis	−1.03	3.62	−0.22	8.08	−0.75	1.39	1.43

**Fig. 9:**
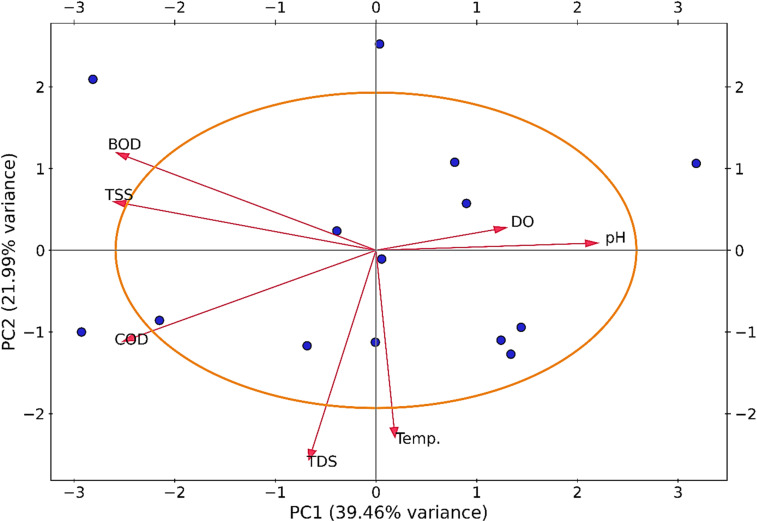
Principal component analysis of the textile effluent parameters.

Fig. 9 shows the PCA, which identified four distinct clusters of wastewater quality parameters based on their similarity: Cluster 1 (BOD and TSS); Cluster 2 (DO and pH); Cluster 3 (temperature); and Cluster 4 (COD and TDS). BOD, TSS, COD, and TDS were the major contributors to PC1 (39.46% variance), whereas DO and pH had the most significant influence on PC2 (21.99% variance). BOD and TSS, COD and TDS, and DO and pH were positively correlated, as indicated by their proximity and similar vector orientation. In contrast, BOD and DO, COD and pH, and TDS and DO showed strong negative correlations. Although temperature showed a weak association with TDS, it remained largely independent of the other parameters.

BOD and TSS formed Cluster 1 due to their strong positive correlation, both representing particulate and organic matter contributions in wastewater. Elevated TSS is often linked to higher BOD, as suspended solids contain biodegradable organic compounds that consume oxygen during microbial decomposition.^78,79^ COD and TDS in Cluster 4 were closely related, reflecting the combined influence of dissolved solids and chemically oxidizable organic matter, which often originate from dyeing auxiliaries, salts, and other industrial chemicals.

Cluster 2 consisted of DO and pH, both of which are critical for aquatic chemical balance. pH affects oxygen solubility and the activity of aquatic organisms, while DO indicates oxygen availability for biological processes. Their strong positive correlation suggests that the conditions under which the optimal pH is maintained also favor high oxygen concentrations.

Temperature, forming Cluster 3, was oriented almost vertically downward, indicating a distinct variation pattern from other parameters. While higher temperatures can enhance mineral dissolution and potentially raise TDS levels, the weak correlation observed in the PCA suggests that the temperature in this dataset was influenced by factors independent of the dissolved solids.

The PCA results also revealed distinct relationships between the effluent characteristics and factory operational performance. Factories equipped with advanced biological or hybrid effluent treatment systems, such as membrane bioreactors (MBR) or bio-chemical hybrid units (*e.g.*, Factories H and K), were positioned closer to the DO–pH cluster, indicating higher oxygenation efficiency and better biological stability in their treated effluent. In contrast, smaller-scale non-green factories, such as A and N, were clustered near the BOD–COD–TSS axis, reflecting elevated organic loads and limited pollutant removal efficiency. This distribution pattern suggests that the type and efficiency of the ETP system, rather than the geographical location, are the key factors influencing wastewater characteristics across the studied factories. Furthermore, the PCA reinforces the strong interdependence of organic load indicators (BOD, COD, TSS, TDS), which together represent the total pollution potential, while DO and pH signify biological stabilization and process efficiency. The grouping of factories in the biplot thus highlights that facilities adopting modern treatment configurations and consistent operational control demonstrate improved effluent quality. Overall, PCA provided deeper insight into the data structure, revealing that treatment technology and management practices are stronger determinants of wastewater performance than production capacity or regional differences.

## 7. Sustainable energy consumption

**Fig. 10:**
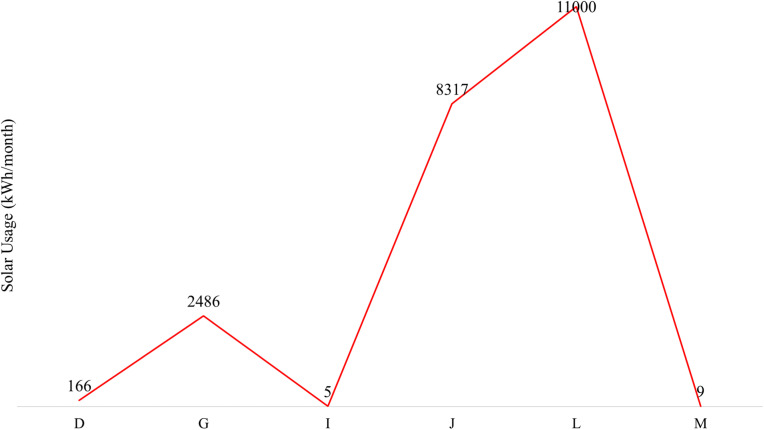
Monthly solar energy consumption (kWh) in the textile factories.

Among the 14 textile industries studied, only six use solar energy, as illustrated in Fig. 10, with monthly consumption ranging from as low as 5 kWh (factory I) to as high as 11 000 kWh (factory L). Increasing solar energy usage in factories with low consumption, like factory I, could significantly reduce their dependence on fossil fuels, leading to lower energy costs and decreased carbon emissions. For example, if factory I scaled its solar use from 5 kWh to even 1000 kWh per month, it could cut hundreds of kilograms of CO_2_ emissions annually, contributing positively to climate change mitigation. On the other hand, factories like L, already using 11 000 kWh monthly, demonstrate how solar adoption can substantially offset conventional energy use, reducing operational costs and minimizing environmental impact on a larger scale. Expanding solar power in textile industries not only enhances economic savings but also offers significant environmental benefits by reducing air pollution, conserving natural resources, and supporting Bangladesh's transition to cleaner, sustainable industrial practices.

## 8. Green technologies in textile processing

**Fig. 11:**
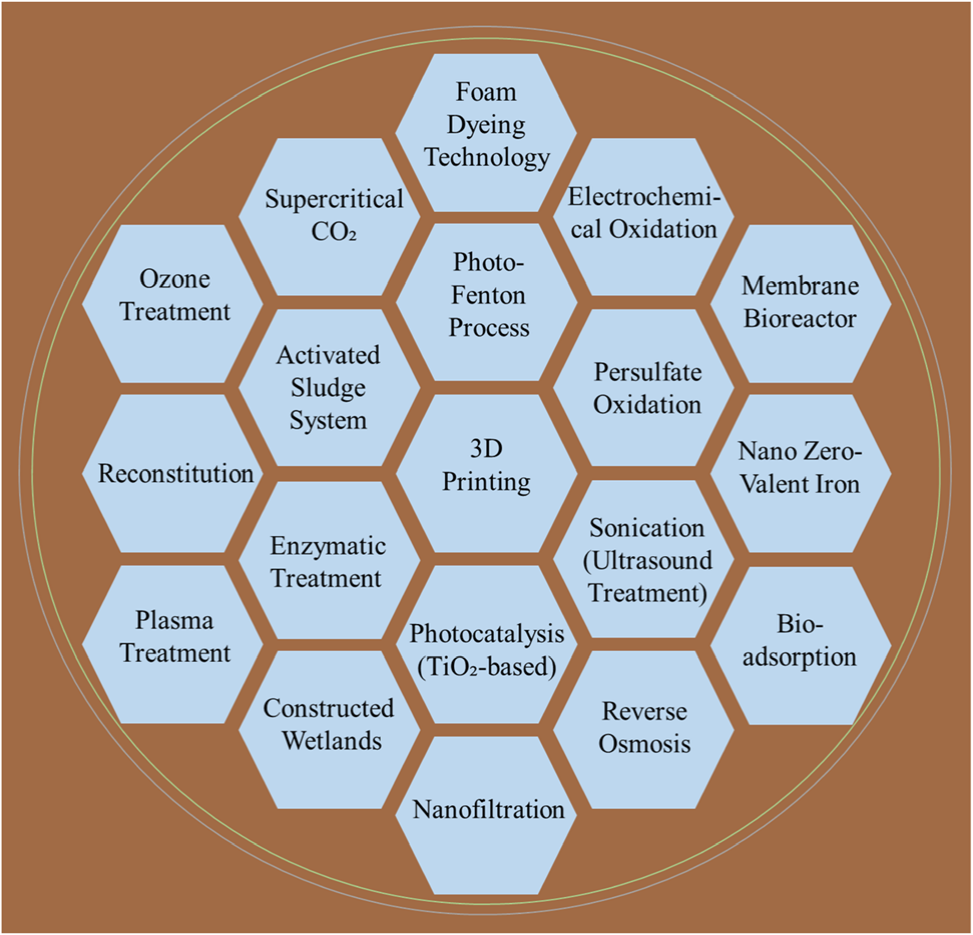
Emerging technologies and methods for sustainable textile processing.

Addressing the environmental challenges of the textile industry necessitates the adoption of advanced technologies that enhance water and energy efficiency while reducing pollution.^80^Fig. 11 illustrates various emerging technologies and methods. Among the emerging innovations, advanced water-recycling systems, low-water dyeing processes, and enhanced filtration techniques play crucial roles in minimizing wastewater volumes and pollutant loads.^81,82^ Additionally, additive manufacturing, particularly 3D printing using materials such as thermoplastic polyurethane and polyethylene, has shown potential for application in reducing water and raw material consumption through precise material application, thereby promoting sustainable production.^83^ Biotechnology also contributes significantly, particularly enzymatic treatments in desizing, scouring, bleaching, dyeing, and finishing, which replace hazardous chemicals with eco-friendly alternatives, improving recyclability and reducing water contamination.^84,85^ Enzymes are equally effective in wastewater treatment.^86–88^ Simultaneously, advanced oxidation processes (AOPs), such as photocatalysis with TiO_2_ under UV light, generate hydroxyl radicals (˙OH) that efficiently degrade persistent pollutants; for example, biological treatment combined with AOPs and 4% H_2_O_2_ achieved 100% decolorization of Remazol Yellow RR dye, alongside 84.88% BOD and 82.76% COD reductions.^89^ Energy-efficient sonication-based AOPs have further improved the removal efficiency of color and refractory compounds.^89,90^ Other biological systems, including activated sludge combined with nanofiltration or reverse osmosis, have yielded colorless effluents with dissolved solid concentrations as low as 196 mg L^−1^. In contrast, persulfate-based oxidation with biological treatment has effectively reduced the COD and BOD.^91^ Moreover, hybrid methods, such as the photo-Fenton process applied pre- and post-nanofiltration, achieved significant COD reduction and color removal efficiencies; however, residuals still hinder direct reuse.^92^ Likewise, coagulation using ferrate, followed by *in situ* ozonation and ceramic membrane filtration, has improved the removal of humic-like substances and biopolymers.^93^ Bio-adsorption techniques using sugarcane bagasse and peanut hulls have achieved dye removal rates up to 74.49% under optimized conditions, supported by FTIR analysis. At the same time, constructed wetlands with vetiver grass proved effective in pollutant reduction in riparian zones.^90,94^ Integrated hybrid systems using FeCl_3_, nano zero-valent iron, and micro-zeolite accomplished high COD (97.5%), TSS (98%), and TN (86.1%) removal efficiencies.^95^ Furthermore, advanced adsorption-membrane configurations and solid-phase extraction using treated agricultural residues have achieved >95% in dye rejection and heavy metal recovery.^96,97^

In addition to wastewater innovations, modifications to the dyeing process play a critical role in conserving energy and water. Techniques that involve low temperatures, short dyeing times, and reduced liquor ratios have gained popularity. For instance, suitable dyeing auxiliaries can enhance dye absorption at ambient temperatures or halve dyeing durations by accelerating absorption rates. However, rapid heating can lead to dye molecule jamming at fibre pores, hindering absorption and necessitating the use of levelling agents to counter the issue.^98^ Sonication has also proven effective in enhancing dye uptake, reducing dyeing time, and improving colorfastness by creating cavitation that breaks down dye aggregates and promotes uniform dye distribution.^99–101^ Chemie Impex introduced Linsegal WRD for wool fibres, significantly reducing dyeing time to 30 minutes by enhancing penetration.^102^ Low-temperature dyeing methods have also been modified by Zhang *et al.*, who treated polyester/cotton fabrics with cyclodextrin to enable dyeing at 70 °C.^103^ Meanwhile, Malik *et al.* utilized triethanolamine to dye cotton at 50 °C, achieving comparable results to traditional methods.^104^ Further, Zhang *et al.* demonstrated that modifying wool fibres with dihydroxyacetone enabled deeper shades and improved UV protection through Maillard reaction dyeing.^105^ Additionally, regular atmospheric polyester dyeing at temperatures below 100 °C was facilitated by various dye-absorption accelerators, like phenol,^106^*para*- and *ortho*-vanillin,^107^ vanillin,^108,109^ and DMSO.^110^ Low-liquor-ratio dyeing has also emerged as a sustainable solution; for example, micelle dyeing using dibutyl maleic acid ester sodium sulfate achieved practical dyeing at a 1 : 5 ratio.^111^ Closed-loop aqueous dyeing methods further promote resource conservation. Tincher *et al.* demonstrated that recycling acid dyebaths for Nylon dyeing saved 35% of energy, 40% of water, and 56% of auxiliaries,^112^ while Hassan and Hawkyard showed that the reuse of ozone-decolorized reactive dyebath was feasible in multiple textile processes.^113^ Other novel methods include foam dyeing, which minimizes or eliminates the need for heating, and transfer dyeing, which utilizes coated paper to transfer dyes to fabrics with minimal water usage. Gel dyeing, where fibres are dyed during their gel phase, enables rapid and efficient dyeing. Hou *et al.* achieved successful acrylic fibre dyeing at 50 °C within 45 seconds, with excellent fastness.^114^ Reverse micellar dyeing, initially developed for cotton and now applicable to other fibres, presents a zero-effluent alternative to traditional exhaust dyeing.^115–117^ Lastly, supercritical CO_2_ (SC-CO_2_) has emerged as a green solvent for waterless dyeing. De Giorgi *et al.* successfully dyed polyester with thiadiazolyl azo dyes in SC-CO_2_ at 80 °C and 24 MPa, achieving results comparable to those obtained in traditional aqueous dyeing at 120 °C, without the need for dispersing agents.^118,119^ Collectively, these advancements highlight a paradigm shift toward environmentally responsible textile processing.

## 9. Limitations of the study

Although this study provides valuable insights into the environmental performance of textile factories in Bangladesh, it is important to recognize certain limitations. The data used were collected during a single month (April 2025), which may not fully capture annual variations in water and energy consumption. Textile production in Bangladesh often fluctuates seasonally due to changes in order volumes, fabric types, and color shades, all of which can significantly influence resource use. Consequently, the results presented here should be interpreted as representative of a specific production period rather than the entire year. Future research should aim to collect multi-month or longitudinal data to account for these variations, thereby improving the robustness and generalizability of findings.

Due to factory confidentiality policies and limited access periods, the data collection period was restricted to April 2025. However, the results still provide valuable insights into the operational characteristics of the studied factories during a representative production cycle.

## 10. Conclusion

This study evaluated water and electricity consumption, solar energy use, and pollution load from effluents discharged by 14 textile industries located in Bangladesh's major textile manufacturing hubs. Large-scale factories were found to consume significantly more water and electricity than smaller ones. A few industries have adopted solar energy to reduce CO_2_ emissions, with some selling excess electricity back to the national grid, accelerating installation cost recovery and generating additional income. Analysis of seven water-quality parameters, including pH, BOD, COD, temperature, TDS, DO, and TSS, showed that discharge values met DoE standards; however, inlet concentrations were often far above permissible limits. PCA revealed strong positive correlations among several parameters, indicating interdependencies that could intensify local surface water pollution if effluents are not adequately treated in ETPs.

This study also highlights the potential environmental, economic, and health risks posed by inadequately treated textile effluents. To address these challenges, the study recommends hybrid or integrated treatment systems combining bioremediation (microbial consortia, phytoremediation), advanced filtration (membrane bioreactors, nanofiltration), and chemical processes (advanced oxidation), offering more sustainable and efficient alternatives to conventional methods, such as coagulation–flocculation and the activated sludge process. Real-time monitoring systems and AI-driven decision-making tools could further optimize treatment performance and ensure compliance with environmental regulations. Policymakers should continue to enforce stricter pollution control to raise awareness among the people and keep the factories environmentally friendly. Furthermore, aligning industrial practices with the Department of Environment (DoE) discharge standards and emerging sustainability policies is crucial for long-term impact. The textile sector in Bangladesh is increasingly guided by national frameworks, such as the Industrial Environmental Compliance Guidelines, the National Energy Efficiency and Conservation Master Plan, and the Bangladesh Climate Change Strategy and Action Plan (BCCSAP). Strengthening water governance through metering, recycling, and zero-liquid-discharge (ZLD) initiatives can support efficient water use. Similarly, adopting renewable energy systems, process heat recovery, and carbon accounting mechanisms can help reduce the industry's carbon footprint. The integration of digital monitoring tools for effluent quality and ETP compliance can further ensure adherence to environmental regulations while promoting resource efficiency across the sector. As this study was limited to a small number of factories in major industrial hubs, future research should include a broader range of regions and facilities, as well as examine socioeconomic factors that can drive cleaner textile production practices.

## Author contributions

Sayam Sayam: conceptualization, methodology, software, formal analysis, resources, writing – original Draft, writing – review & editing, visualization, supervision, project administration, data curation; Nayan Das: writing – original draft, data curation; Sumiya Akter: writing – original draft; and Joya Saha, Asish Sarker, Ayanshu Sen, Habibullah, Gulger Ahmed Sajib, Infanul Haque, Md. Bayezid Mia, Md. Omar Faruk, Sanchita Fatema, Md. Kayes Munshi, Pias Chandra Paul, Md. Iftekhar Haider: data curation.

## Conflicts of interest

There are no conflicts to declare.

## Data Availability

The data supporting the findings of this study are available from the corresponding author upon request.
